# Movement Termination of Slow-Wave Sleep—A Potential Biomarker?

**DOI:** 10.3390/brainsci14050493

**Published:** 2024-05-13

**Authors:** Yvonne Höller, Stefanía Guðrún Eyjólfsdóttir, Matej Rusiňák, Lárus Steinþór Guðmundsson, Eugen Trinka

**Affiliations:** 1 Faculty of Psychology, University of Akureyri, 600 Akureyri, Iceland; ha170343@unak.is (S.G.E.); mar3@unak.is (M.R.); 2 Faculty of Social Studies, Masaryk University, 601 77 Brno, Czech Republic; 3 Faculty of Pharmaceutical Sciences, University of Iceland, 102 Reykjavík, Iceland; larussg@hi.is; 4 Department of Neurology, Neurointensive Care and Neurorehabilitation, Christian-Doppler University Hospital, Paracelsus Medical University, Centre for Neuroscience Salzburg, Member of the European Reference Network, EpiCARE, 5020 Salzburg, Austria; 5 Neuroscience Institute, Christian-Doppler University Hospital, Centre for Cognitive Neuroscience, 5020 Salzburg, Austria

**Keywords:** slow-wave sleep, sleep movements, sleep EMG, epilepsy, Sertraline

## Abstract

The duration of slow-wave sleep (SWS) is related to the reported sleep quality and to the important variables of mental and physical health. The internal cues to end an episode of SWS are poorly understood. One such internal cue is the initiation of a body movement, which is detectable as electromyographic (EMG) activity in sleep-electroencephalography (EEG). In the present study, we characterized the termination of SWS episodes by movement to explore its potential as a biomarker. To this end, we characterized the relation between the occurrence of SWS termination by movement and individual characteristics (age, sex), SWS duration and spectral content, chronotype, depression, medication, overnight memory performance, and, as a potential neurological application, epilepsy. We analyzed 94 full-night EEG-EMG recordings (75/94 had confirmed epilepsy) in the video-EEG monitoring unit of the EpiCARE Centre Salzburg, Austria. Segments of SWS were counted and rated for their termination by movement or not through the visual inspection of continuous EEG and EMG recordings. Multiple linear regression was used to predict the number of SWS episodes that ended with movement by depression, chronotype, type of epilepsy (focal, generalized, no epilepsy, unclear), medication, gender, total duration of SWS, occurrence of seizures during the night, occurrence of tonic-clonic seizures during the night, and SWS frequency spectra. Furthermore, we assessed whether SWS movement termination was related to overnight memory retention. According to multiple linear regression, patients with overall longer SWS experienced more SWS episodes that ended with movement (t = 5.64; *p* = 0.001). No other variable was related to the proportion of SWS that ended with movement, including no epilepsy-related variable. A small sample (n = 4) of patients taking Sertraline experienced no SWS that ended with movement, which was significant compared to all other patients (t = 8.00; *p* < 0.001) and to n = 35 patients who did not take any medication (t = 4.22; *p* < 0.001). While this result was based on a small subsample and must be interpreted with caution, it warrants replication in a larger sample with and without seizures to further elucidate the role of the movement termination of SWS and its potential to serve as a biomarker for sleep continuity and for medication effects on sleep.

## 1. Introduction

Slow-wave sleep (SWS) is said to be the most important part of the night, as the most restorative sleep stage, and its duration is linked to reported sleep quality [1]. SWS is part of the non-rapid eye movement (NREM) sleep, corresponding to stages 3 and 4 in the classification of Rechtschaffen and Kales [2] and stage N3 in the new AASM (American Academy of Sleep Medicine) manual [3]. Healthy individuals spend 10 to 25% of their total sleep time in SWS [4].

It was suggested that muscular activity events during sleep might be an important correlate of sleep continuity and, as such, subjective and objective sleep quality [5]. In healthy populations, movements during sleep are more likely to occur at the beginning and end of NREM sleep [5]. These sleep movements can be detected as EMG events in the EEG [5]. Research so far suggests that sleep movements that coincide with the end of an SWS phase are a naturally occurring phenomenon rather than representing a disturbance of SWS [5]. From studies that aimed at disrupting SWS through stimulation, it is known that the duration of SWS can be reduced without impacting the overall sleep architecture [6] or duration [7]. These studies used acoustic stimulation to induce arousals and possibly brief awakenings [8]. Selective suppression of SWS reduces delta power during SWS [8] but during the recovery of SWS, delta power was found to be higher, suggesting a homeostatic process [9,10]. Under this assumption, it is only plausible that the homeostasis of SWS governs the duration and, thus, physiological end of SWS through internal cues. The muscular correlates of movement at the end of SWS are present in undisturbed sleep [5] and are, thus, likely to reflect such an internal cue to end the SWS phase. We might even speculate that movement at the end of SWS marks the homeostatic termination of SWS. However, no systematic investigation of the factors that are potentially associated with the phenomenon of the movement termination of SWS has been conducted so far. The aim of the current study was to explore the potential value of the movement termination of SWS as a biomarker. We chose epilepsy as an exemplary application area, because of the close relationship between epilepsy and sleep. Epilepsy affects the sleep architecture [11], and if deep sleep movement terminations in epilepsy patients are a pathological interruption, they could play a significant role in the complex interplay between epilepsy and SWS. On the other hand, clinical features such as interictal epileptiform activity or medication could disturb the homeostasis of SWS, and movement at the end of SWS could be a marker for a disturbed homeostasis. In this context, it is valid to ask whether the duration of SWS could be related to the occurrence of muscular activity at the end of the SWS episode.

When investigating the research question whether the movement termination of deep sleep could be a biomarker for individual characteristics and clinical features, and whether it is of particular interest for patients with epilepsy, a multitude of factors need to be considered.

Firstly, even in healthy populations, only a certain proportion of SWS end with a muscular artefact [5], but this proportion is documented only in a small sample. Therefore, it is of interest whether the number of SWS ending with movement is comparable between patients with an epilepsy diagnosis and people without recurrent seizures. Furthermore, the type of seizures (focal vs. generalized) might play a role.

Secondly, sleep is related to the duration of epilepsy and, in this respect, the age of onset of the condition [12]. In more general terms, the sleep architecture changes considerably with age, and especially, SWS decreases linearly at 2% per decade up to the age of 60 years [13]. Thus, it is of interest whether the movement termination of SWS is related to age.

With respect to sleep and age, the chronotype is an important third factor. Younger people are more likely to be an evening chronotype, and overall, evening chronotypes are more likely to suffer from mental health disorders, including depression and insomnia [14]. Especially in standardized sleep environments, the chronotype plays a significant role because it impacts the sleep onset latency. Therefore, evening chronotypes have a shorter total sleep duration [15], especially under conditions with regulated, usually early awakening times.

Fourthly, sex affects the sleep architecture. Women have overall better sleep quality compared to men, especially higher sleep efficiency, but also more sleep-related complaints and a complex effect of hormones on sleep [16]. Furthermore, the effect of gender interacts with age. The reduction of SWS is stronger in men than in women [13].

Fifth, depression affects sleep and vice versa [17], and this is also true for patients with epilepsy [12]. Depression is linked to the changes in sleep continuity and impaired NREM sleep, with sleep-EEG biomarkers being useful for the diagnosis, prognosis, and prediction of the therapy response in depression [17]. In this respect, the interaction between sleep and psychoactive medication, especially antidepressants [17], needs to be considered.

This leads to the sixth important factor, medication. Antidepressants are only one type of drug that impacts sleep, as many other drugs impact sleep, especially antiseizure drugs [18]. However, it depends on the type of drug whether the effect might be an increase or decrease in SWS duration [18].

Having considered the most important factors that might interact with the presumed biomarker, i.e., the movement termination of SWS, several consequences should be investigated to characterize the potential of the marker even further. We limited the present study to two domains: memory retention and SWS frequency spectra.

In healthy populations, SWS benefits declarative memory formation [19]. Deprivation from SWS was also found to decrease the counting performance [20], slow-wave activity correlates with the performance in texture-perceptual learning [21] and visuomotor coordination [22]. Among the patients with epilepsy, the effects of sleep on memory retention seem to be comparable to those found in healthy controls [23]. However, nocturnal seizures [24] and sleep-related interictal epileptiform activity [25,26,27,28] impact sleep and, consequently, cognitive function [29].

SWS frequency spectra are related to pressure for SWS, with higher delta and theta power after the deprivation of SWS [9,10]. EEG alpha asymmetry is a marker for various brain disorders, and it is stable across sleep and wake states, with the highest amplitudes during SWS [30]. Furthermore, the variation in sleep spindle activity can be documented by reference to the power density in the range of 12–15 Hz [1,31]. Sleep spindles are related to several of the above-mentioned factors, such as gender, as they are modulated by the menstrual cycle [32], and most importantly, memory [33].

In the present study, we examined the movement termination of SWS segments as a potential novel biomarker that could contribute to the diagnosis of neurological disorders and whether the occurrence of movement at the end of SWS is related to the epilepsy diagnosis and epilepsy type, duration of deep sleep, age, age of onset, gender, depression, chronotype, drug load against a defined daily dose of antiseizure medication and psychoactive medication, as well as type of drug. Furthermore, we investigated the relation between the movement termination of SWS and SWS power spectra, as well as the overnight verbal, episodic, and procedural memory retention.

## 2. Materials and Methods

### 2.1. Ethics

The ethical commission of the Region of Salzburg, Austria approved the study protocol (approval nr. 415-E/1755/20-2016). The study was carried out in compliance with the Declaration of Helsinki. All the patients signed written informed consent forms.

### 2.2. Setting and Recruitment

The study took place at the Epilepsy Monitoring Unit (EMU) of the Department of Neurology, member of EpiCARE, Christian Doppler University Hospital of the Paracelsus Medical University in Salzburg, Austria. The unit includes a patient room with four beds and a monitoring room, where trained staff monitor the patients’ electroencephalogram (EEG) and video. The monitoring room is separated from the patient room with a window, so staff can also directly oversee the patient room. The patients’ daily rhythm in the epilepsy monitoring unit follows fixed times of lights on/off and meals. Lights are turned on in the morning (06:30–07:00), after which breakfast is served (07:00–07:30); then, lunch (11:30) and dinner (16:30) follow. Finally, the lights are turned off in the evening (22:00–00:00). During the monitoring period, the dosage of antiepileptic drugs is commonly tapered to provoke seizures. For the same purpose, the patients undergo sleep deprivation during the third night to provoke seizure occurrence. A stay at the EMU typically lasts from Monday until Friday. Most patients are admitted to the EMU to either clarify whether suspected events represent seizures, to facilitate a more detailed diagnosis of their epilepsy syndrome, to collect data for presurgical evaluation, or to monitor antiseizure medication changes or efficacy. The details of the procedures at the EMU in Salzburg have been published previously [34,35,36]. Since the purpose of the study within which the present data were collected was to investigate the effect of seizures on memory, every week, we chose the patient among the four admitted patients who had a high likelihood of receiving an epilepsy diagnosis and the occurrence of seizures based on existing patient history. The patients were furthermore selected based on age (between 18 and 75 years), fluency in the German language to be able to perform the memory tests, adequate intellectual ability to give informed consent, and no existing diagnosis of neurological diseases of a degenerative nature. As a first step, informed consent was obtained following admittance on Monday morning. Then, we recorded their demographical information, and the patients were tested for depression using the German version of Beck’s Depression Inventory [37]. Furthermore, the German version of the Morningness–Eveningness Questionnaire (DMEQ) was conducted [38] to control for a poor mismatch between the patients’ own biorhythm and the daily rhythm imposed at the hospital. An initial medical examination followed, and then, the patients were prepared for the long-term EEG monitoring performed by the medical technicians. Continuous monitoring involved the requirement to stay in bed as much as possible for reasons of safety. The patients could disconnect the fixed EEG recording system to use the bathroom. The system’s mobile module allows for continued recording during those periods.

### 2.3. Memory Testing Procedure

The memory testing procedure is described in more detail in a previous publication [39]. We tested the participants in the evening and in the morning throughout the week using three different tasks, accounting for procedural memory, verbal memory, and episodic memory. In the evening, the participants learned the material and performed an immediate recall. In the morning, a delayed recall was performed. To be able to test the participants every night, we used new material on every day of the testing.

In the procedural memory task (see [40] for more details) the participants learned to type a sequence on the computer keyboard as fast and as accurately as possible within 12 trials of 30 s interspaced by 30 s of breaks. As an immediate learning outcome measure, we averaged the number of correctly typed 3-element sequences within the last three trials. Recall in the morning consisted of only 4 trials, where we took an average again over the last 3 trials. We calculated the ratio of triplets between the morning and the evening to obtain a measure of overnight retention of procedural memory.

In the verbal memory task (see [39] for more details), the participants learned 60 pairs of nouns that were standardized for emotional valence and arousal using the Berlin Affective Word List [41,42]. Half of the words were related, and half were unrelated. To facilitate active encoding, during learning, the participants were asked to rate whether the pairs of words were related or unrelated. During the recall, only the first word was presented, and the participants were required to name the second word. The outcome measure was the number of correctly remembered words. We calculated the ratio of correctly remembered words at the delayed recall in the morning vs. the correctly remembered words at the immediate recall in the evening.

In the episodic memory task (see [43,44] for more details), we asked the participants to navigate through a virtual town, created in UNITY (Unity Technologies ApS, Engine Version 5.3.5fl, Unity3d.com). The town included 10 turns (left or right) that contained scenes with at least two elements that could be remembered. After navigating through the town using the keyboard’s cursor keys, the participants performed an immediate recall. They were first asked to name all the elements that they remembered (WHAT outcome of memory and then, to describe these elements (DETAILS outcome of memory). Furthermore, the participants had to judge for each remembered element whether they had seen it at the beginning (first 3 scenes), middle (middle 4 scenes), or end (last 3 scenes) in the town (WHEN outcome of memory). For each element, they were also asked whether they had seen it to their left or right (EGOCENTRIC WHERE outcome of memory) and whether other elements in the scene were to the left, right, in front, or behind the element in question (ALLOCENTRIC WHERE outcome of memory). The delayed recall in the morning proceeded analogously. To obtain one memory index, we added up all the correct memories across the examined memory outcomes and calculated the ratio between this sum from the delayed recall in the morning vs. the immediate recall in the evening.

### 2.4. Drug Load

Like in a previous publication [43], we determined the drug load on the day of recruitment and on the day preceding the analyzed night. For each drug that had a known effect on brain activity, we calculated the drug load as the ratio between the dose taken on that day and the defined daily dose. Finally, we added the loads over all the drugs taken on that day, separately for psychoactive drugs and antiseizure medication (ASM) [45].

Additionally, we determined for each patient the type of drug taken and grouped patients into those taking the individual drugs, or groups of individual drugs. The groups of drugs considered were ASM, selective serotonin reuptake inhibitors (SSRIs), other antidepressant medications, Benzodiazepines, and antipsychotic medication.

### 2.5. EEG Recording

EEG recordings were obtained by using the hardware SystemPlus Evolution, SD LTM 46 Express Amplifier (Micromed S.p.A, Mogliano, Italy). EEG recordings lasted from the day of admission (usually Monday) until the day of discharge from the EMU (usually Friday). The recording was conducted using 29 standard electrodes placed according to the 10–20 system at a sampling rate of 1024 Hz and on-line filtering using a 0.1 Hz high-pass and 50 Hz notch filter. Location Fpz served as the ground and Oz as the reference electrode. Impedances were kept below 10 kΩ on the day of setup, and data quality was monitored daily to adjust it, if necessary, such that this quality standard was maintained. Additional electrodes measured the differential electrocardiogram, electromyogram at the chin, and horizontal electrooculogram, where the latter two were mounted only in the evening and removed in the morning.

### 2.6. Data Selection

We aimed at including the data from the second night because of the well-known bias resulting from habituation of the patient to the recording environment during the first night [46]. The third night was usually not elective because of the common exposure to sleep deprivation. However, if we could not identify SWS segments during the second night or if the patients had undergone sleep deprivation already during the second night, we considered the third night for analyses. If neither the second nor the third night was eligible, the patients were excluded from further data analysis.

### 2.7. Seizure Identification

Seizure monitoring is part of the tasks of the specialist staff working at the EMU. During the day shifts, the medical technical nurses pre-screened the recordings and marked the beginnings of suspicious events for further investigation. Seizures were also monitored in real time based on EEG and behavioral cues. When a seizure occurred, an ictal test was conducted by the present medical staff. The procedure evaluated the responsivity, sensation and perception, consciousness, memory, and basic cognitive skills as per a standardized testing protocol [47]. All the events that were possibly seizures were further examined by a neurologist to confirm the markings.

Within the present project, a clinically trained EEG technician performed a further detailed scoring of all the seizures in the EEG for all included patients and classified the seizures into tonic-clonic seizures and other seizures, with a specific emphasis on distinguishing nocturnal seizures with post-seizure slow-wave activity from SWS. Based on this information, we also compared the time of seizure onset to the time of onset of SWS to avoid misinterpretation of SWS episodes that ended with a seizure.

### 2.8. Slow-Wave Sleep Staging

All the night files were extracted from the clinical system and imported to the software Brain Vision Analyzer 2.0 (Brain Products GmbH, Gilching, Germany). We followed the manual of Rechtschaffen and Kales [2] for sleep staging. To identify SWS, corresponding to stages 3–4 in the manual of Rechtschaffen and Kales [2] and stage N3 in the new AASM (American Academy of Sleep Medicine) manual [3], a trained researcher scrolled through the EEG in 20 s epochs as recommended for fragmented sleep [48] and as performed in a previous study regarding EMG activity during sleep [5]. A segment was identified as SWS if slow waves of a frequency below 2 Hz with a high amplitude were present and synchronized activity occupied at least 50% of an epoch [49]. During this procedure, markers were set at the beginning and end of each SWS segment. These markers were reviewed by an experienced EEG-researcher and adjusted if necessary. We extracted the total duration of SWS by summing up the duration of each SWS segment during the examined night for a patient. All the segments were reviewed by an independent, trained researcher who scored their ending as an ending with an event that involved a large muscular artefact. These segments were counted as those ending with movement.

### 2.9. Spectral Analysis of EEG-SWS

All SWS episodes were divided into epochs of 4 sec, and each epoch was transformed using the Fast Fourier Transform (resolution 0.25 Hz, non-complex output in microvoltage, half spectrum). In the first analysis, we averaged all the power spectra across all the 4 s epochs of all the SWS episodes. In the second analysis, we extracted the last 20 s of each SWS segment and calculated the same Fast Fourier Transform for that segment. For both of these analyses, the power density was calculated for the frequency bands delta (0.5–4 Hz), theta (5–7 Hz), alpha (8–12 Hz), and sigma (13–15 Hz) as unsigned, rectified values of mean activity for each band. These values were averaged across the regions of interest: frontal left (F3, F7, F11), frontal right (F4, F8, F12), central left (C3), central right (C4), temporal left (T7, T11), temporal right (T8, T12), parietal left (P3, P7, P11), parietal right (P4, P8, P12), occipital left (O1), and occipital right (O2).

### 2.10. Statistics

All the statistics were carried out using R 2022-06-23 in R-Studio version 2022.02.3+492 [50]. The descriptive statistics were given as means and standard deviations (SD) for the metric variables, median, and range for ordinal variables, and counts for categorical variables.

The metric variable comparisons between groups were performed by using Pearson’s independent samples *t*-test.

The proportions of groups falling into categories (i.e., gender x experience of any movement termination of SWS) were compared using a chi-squared test. For the additional analysis of a very small sample taking a medication, we performed a permutation test using the perm.tests function from the R package AUtests (version 0.99). This permutation test computes a test statistic for the observed data by generating all the datasets with the same total number of exposed subjects, then adding up the probabilities of those datasets which give more extreme test statistics than the observed data.

We performed Spearman correlations between the variables of interest (age) and the proportion of SWS ending with movement among all the SWS episodes during the examined night.

The candidate predictor variables of age, age of onset, gender, the duration of deep sleep, depression as measured by the BDI, chronotype as measured using the DMEQ, drug load against defined daily dose of antiseizure medication and psychoactive medication, and epilepsy type (focal, generalized, no epilepsy, unclear) were modelled with respect to their potential relation to the number of movement terminations of SWS during the respective night using multiple linear regression (R function “lm”).

We also performed linear multiple regression model estimation to test whether the frequency spectra during all-night SWS in specific regions of interest were predictive for the number of SWS episodes that ended with movement. For each frequency band, one model was calculated (delta, theta, alpha, and sigma). Additionally, we performed a logistic regression (based on R function “glm” and the “logit” option for the binomial family of models) where we predicted for all the single SWS episodes whether they ended with movement or not based on the power spectra in the regions of interests, separately for the same frequency bands. Since this analysis was not based on independent data because of the varying number of SWS episodes per patient, we performed the same analysis again only for the first SWS episode per night.

We performed linear regression models for the verbal, episodic, and procedural memory overnight retention ratios for a subset of patients as not all patients underwent these memory tests. The predictor variables included depression (BDI score), the chronotype (DMEQ score), type of epilepsy (focal, generalized, no epilepsy, unclear), drug load of antiseizure medication, drug load of psychoactive medication, gender, total duration of SWS, occurrence of seizures during the night, occurrence of tonic-clonic seizures during the night, and the proportion of SWS episodes that ended with movement.

## 3. Results

### 3.1. Sample

In total, 106 patients were recruited in the Epilepsy Monitoring Unit (EMU) Salzburg between February 2016 and June 2018, usually one per week. Four patients were not further analyzed because they underwent invasive recordings, three patients were excluded because of incomplete EEG data and during the night recordings of five patients, no SWS was found, neither during night 2 nor night 3. The details of the final sample of 94 patients are given in the Supplementary Data File. In this final sample, there were 48 women (mean age = 31; SD = 12.17) and 46 men (mean age = 36.26; SD = 17.49). We analyzed night two for 84 patients, while for the other 10, we analyzed night three. There were 11 patients who were left-handed. After the 5-day video-EEG monitoring, for 75 patients, a diagnosis of epilepsy was confirmed or established, for 11 patients the conclusion was that they did not have epilepsy, and for 8, the diagnosis was unclear. In the sample of patients with an established epilepsy diagnosis, the seizure type was in most cases focal (n = 59). In 14 cases, seizures were generalized, and in two cases, it was undetermined whether the seizure type was focal or generalized. The diagnoses in this sample of patients without an epilepsy diagnosis were transient global amnesia (1), spastic hemiparesis (1), panic attacks (2), transient disorder of consciousness of unknown etiology (1), convulsive syncope (1), migraine (2), cardiovascular disorder (1), pneumonia (1), and spinal fracture (1). The localization of focal seizures was determined to be temporal left in 15 cases, 12 cases had temporal right localization, 2 cases had temporal-bilateral localization, 8 cases had frontal left localization, 1 had frontal right localization, 6 had frontal bilateral localization, and 6 more had other frontal localization, two patients had their seizures occipitally localized, four had their seizures parietally localized, and the others had an unclear localization. The age of seizure onset was on average at the age of 22.75 years (SD = 15.12) for patients with focal seizures and at the age of 17.08 (SD = 10.06) for patients with generalized seizures. Magnetic resonance imaging was available at the time of inclusion in the study from 40 patients with focal epilepsy, of which 25 showed structural abnormalities. There were 14 patients who had experienced status epilepticus in the past.

The chronotype was assessed in all but 6 of the 94 participants. There were 51 patients who were classified as neutral types, 13 were rather evening types, 14 were rather morning types, four were clearly evening types, and another four were clearly morning types.

Depression was assessed in all but 11 patients. In 42 patients, no symptoms of depression were detected (BDI score < 10), 15 showed minimal symptoms (BDI score ≥ 10), 17 showed mild depression (BDI score ≥ 14), 5 showed moderate depression (BDI score ≥ 20), and 4 showed severe depression (BDI score ≥ 29).

### 3.2. Medication, Seizures, and Slow-Wave Sleep

On the day of admission, there were 24 patients who did not take any ASM, 42 took one type, 21 took two types, and 7 took three types. On the day before the assessed night, there were 41 patients who did not take any ASM, 31 took one type, 21 took two types, and 1 took three types. Among all the patients on ASM on the day before the assessed night, the average drug load was 1.13 (SD 1.10). Regarding psychoactive medication, on the day of admission, 77 took none, 10 patients took one type, 3 took two types, 3 took three types, and 1 took four types of psychoactive medication. On the day before the examined night, 76 took none, 10 patients took one type, 4 took two types, 3 took three types, and 1 took four types of psychoactive medication. Among all the patients who took psychoactive medication, the average drug load was 1.37 (SD 1.34) on the day before the experimental night.

During the assessed night, there were 14 patients with focal epilepsy who experienced at least one seizure, of which three were tonic-clonic seizures. Only one patient with generalized epilepsy experienced a seizure during the assessed night, which was a tonic-clonic seizure.

SWS during the examined night lasted on average for 71.96 min (SD = 39.17).

### 3.3. Counts of the Movement Termination of SWS

**Figure 1:**
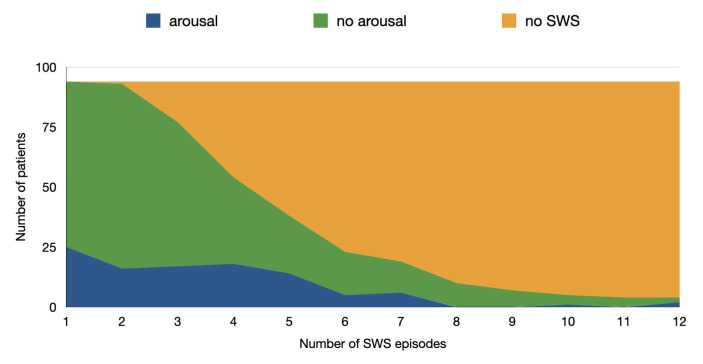
Distribution of the number of SWS segments that ended with movement over the course of SWS segments in the sample of n = 94 patients. The *y*-axis represents the number of patients (max n = 94) and the *x*-axis represents the number of SWS segments.

In a total of 431 SWS segments, 104 ended with movement. Figure 1 shows the distribution of the movement termination of SWS segments over the number of SWS segments found per patient. Because of the fragmented sleep, some patients had a high number of short SWS segments.

**Figure 2:**
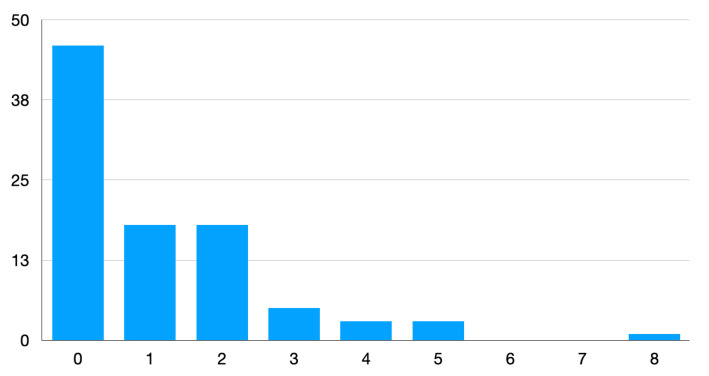
Histogram of the number of movement terminations of SWS per investigated patient in the overall sample, regardless of epilepsy diagnosis. The *y*-axis shows the number of patients; the *x*-axis shows the number of SWS segments that ended with movement.

Figure 2 shows the histogram of the movement termination of SWS per patient, regardless of their epilepsy diagnosis. Among the patients with confirmed epilepsy, 40 had no occurrences of the movement termination of SWS, while 35 patients with epilepsy showed at least one such episode. Among the 11 patients who were not diagnosed with epilepsy, there were 3 where no movement termination of SWS occurred, while in 8, some SWS episodes ended with movement. Among the patients with unknown focal or generalized epilepsy diagnosis there were three without any SWS episode ending with movement while five had at least one SWS episode that ended with movement.

Since the number of SWS segments varied from patient to patient, we calculated the percentage of SWS segments that ended with movement per patient. Among all the patients that demonstrated at least one such episode, the mean proportion of SWS segments ending with movement was 46.33% (range 11.11% to 100%, SD = 24.53%).

### 3.4. Duration of SWS That Ended with Movement

We compared the duration of SWS episodes that were terminated by movement (mean duration = 19.05 min; SD = 16.41) to those that did not end with movement (mean duration = 14.49 min; SD = 13.38) using a simple *t*-test. SWS episodes that did not end with movement were significantly shorter than those that did end with movement (t = −2.57; *p* = 0.011). Since this test included multiple measurements per patient, the values were not independent, and the result must be interpreted with caution. Comparing individual segments (first, second, third, etc.,) of SWS during the night to each other by their ending with or without movement did not yield any statistical difference.

### 3.5. Patient Characteristics Associated with the Number of Movement Terminations of SWS

The occurrence of movement at the end of SWS did not depend on the diagnosis of epilepsy according to a chi-square test (chi-square (1) = 1.67; *p* = 0.197).

The proportion of SWS terminated by movement did not correlate with age (spearman rho = −0.04; *p* = 0.688). A chi-square test assessing the probability of the occurrence of any number of SWS episodes terminated by movement being dependent on gender revealed no effect (chi-square (1) = 0.67; *p* = 0.412).

We performed a linear multiple regression model estimation to test whether age, gender, the duration of deep sleep, depression as measured by the BDI, the chronotype as measured using the DMEQ, the drug load against the defined daily dose of antiseizure medication and psychoactive medication, and in terms of epilepsy, the age of epilepsy onset and epilepsy type, would determine how many SWS episodes were terminated by movement per night.

We found that the only significant predictor was that the likelihood of the occurrence of movement at the end of SWS was higher with a longer total duration of deep sleep (see Table 1), which might simply be explained by the situation that more deep sleep allows for more movement to occur, as patients who do not sleep much have also fewer chances to experience SWS episodes with movement.

The same result was obtained when restricting the sample to the patients with confirmed epilepsy diagnosis. When using the percentage of SWS episodes that ended with movement instead of the number of SWS episodes that ended with movement in the model, the factor total duration of SWS was no longer significant.

### 3.6. SWS Frequency Spectrum Characteristics Associated with the Number of Movement Terminations of SWS

The results of the linear regression models for any frequency band and the regions of interest are summarized in Table 2. Note that Table 2 shows only results with a *p* < 0.100, not all the regions for the four examined frequency ranges.

There were no significant predictors for the number of SWS episodes that ended with movement when the overall power spectra of SWS served as predictors. Trends (significant before, but not after correcting for multiple comparisons) were observed for parietal left alpha and sigma, as well as parietal right sigma activity.

### 3.7. Overnight Memory Retention and the Number of SWS Episodes That Ended with Movement

For the 56 patients who completed the procedural memory testing within the fingertapping task, their overnight memory retention was significantly better in the group of patients without epilepsy (estimate: 2.30; SE = 0.676; t = 3.402; *p* = 0.001). No other significant relationship was found for procedural memory.

For the 65 patients who completed the verbal memory testing before and after the investigated night, the overnight memory retention was solely significantly worse when the patients experienced tonic-clonic seizures during the night (estimate = −0.37; SE = 0.15; t = −2.407; *p* = 0.020; significant only before correcting for the multiple testing).

For the 57 patients who completed the episodic memory test, a better episodic memory ratio was predicted solely by a shorter total duration of SWS (estimate = −0.00005; SE = 0.00002; t = −2.12; *p* = 0.040; significant only before correcting for multiple testing).

In sum, the overnight memory retention was not related to the number of SWS episodes that were terminated by movement.

### 3.8. Relation between the Ending of SWS Segments and Medication

While the defined daily dose of drugs was found to have no relation to the occurrence of SWS episodes that ended with movement, we additionally analyzed whether patients who took specific drugs that knowingly influence SWS would follow a specific pattern. Since the number of patients for some drugs was very small, we limited this analysis to all the drugs where the group of patients who took the respective drug was at least n = 4. In Table 3, we provide the means and standard deviations for the percentage of SWS episodes that ended with movement, separately for the group that we defined by taking a specific drug, or group of drugs, and all the other patients. We additionally provided statistics (*t*-tests) for this group comparison as well as the group who took that specific drug, or group of drugs, as compared to the group of n = 35 patients who did not take any ASM or psychoactive medication. This group had a mean percentage of SWS that ended with movement of 0.20 (SD = 0.28). Specific ASM drugs not represented in Table 3 were Ethosuximide (n = 1), Eslicarbazepinacetate (n = 1), Lamotrigine (n = 3), Zonisamide (n = 2), Perampanel (n = 1), Pregabaline (n = 1), and Topiramate (n = 2). Antipsychotic drugs not represented in Table 3 were Aripiprazole (n = 2), Quetiapine (n = 2), Clozapine (n = 1), and Risperidone (n = 1). Benzodiazepines not represented in Table 3 were Clonazepam (n = 1), Lorazepam (n = 1), Triazolam (n = 1), and Clobazam (n = 1). Selective serotonin reuptake inhibitors (SSRIs) with too small sample sizes were Citalopram (n = 3), Escitalopram (n = 1), and Fluoxetine (n = 1). In the group of other antidepressants not included in Table 3 were Trazodone (n = 3), Duloxetine (n = 1), Venlafaxine (n = 2), and Mirtazapine (n = 2). Finally, for the group of opioids, we could not analyze Hydromorphone (n = 1) because of the small number of patients taking this drug. None of the medications apart from Sertraline showed a significant effect on the percentage of SWS that ended with movement. None of the patients who took Sertraline experienced the movement termination of SWS during the assessed night; therefore, this value differed significantly from the group of patients not taking Sertraline, as well as from the group not taking any ASM or psychoactive drug. Because of the small sample taking this drug, this result must be interpreted with caution. In order to address the possibility that this was an effect by chance, since many participants did not show SWS episodes that were terminated by movement, we performed a permutation test. In this test, we compared the ratio of the patients experiencing at least one SWS episode that ended with movement vs. those who did not have any SWS terminated by movement among the patients not taking Sertraline but taking any other medication (32:23) with the same ratio among the patients taking Sertraline (0:4). According to this test, the effect of Sertraline was significant (*p* = 0.039).

To document the overall effect of the assessed drugs on SWS, the same analysis was conducted for the total duration of SWS (Table 4).

## 4. Discussion

The present study examined the movement termination patterns of SWS episodes in people with and without epilepsy, and the potential associations with various factors including the type of seizures, patient demographics, medication intake, EEG power spectra, and memory retention. The findings support the view that the muscular activity at the end of SWS episodes are not related to any clinical aspect of epilepsy assessed in this study and are therefore unlikely to serve as a biomarker for epileptic pathology. However, several interesting observations could be made that warrant further investigation of SWS termination by movement as a potential biomarker.

### 4.1. Movement Patterns at the End of SWS: Proportion and Duration of SWS

One of the key observations was that nearly half of all SWS episodes examined in the study terminated with movement. This high proportion warrants further research to gain a better understanding of the mechanisms governing movement during sleep, particularly in populations with neurological conditions.

Although relevant for the sleep architecture, the demographic factors such as age and gender did not exhibit significant correlations with the occurrence of SWS terminations with movement. Also, there was no relation between the SWS termination patterns and overnight memory retention. The dynamics of SWS termination were not related to any of the assessed factors in this study, except for the duration of SWS overall, and possibly the duration of individual SWS episodes. The most plausible interpretation is, therefore, that the movements at the end of SWS are a sporadic but naturally occurring physiological marker for the end of SWS.

SWS segments that ended with movement were significantly longer compared to those without movement termination, indicating a potential role of sleep depth in influencing termination patterns. However, caution is warranted in interpreting this result due to the lack of independence in the employed statistical analysis. Instead, the total duration of deep sleep emerged as the sole significant predictor for the overall number of movement terminations of SWS, suggesting an interplay between the sleep architecture and movement dynamics. While the most plausible interpretation of this finding is that a longer duration of SWS gives rise to more chances for movement during SWS, this finding could, as well, indicate a relation between the urge to change bodily position and the duration of (deep) sleep. In a study with eleven young adults, different kinds of sleep disturbances were tested, i.e., brief awakenings, the requirement to make a quarter-body turn, and a disturbance in the ongoing EEG change [51]. While these three conditions differentially impacted sleep, with the most disturbance following the awakening condition and the least following the EEG change condition, it was found that the restorative function of sleep is equally impaired by any periodic change in ongoing EEG. Thus, if the presented patterns of movement at the end of SWS were an interruption, we would have expected a shorter SWS. In contrast, we related a longer duration of SWS to more SWS episodes ending with movement. The implications of this finding extend beyond epilepsy research and might trigger further investigation into the mechanisms governing the relationship between sleep length and movement propensity.

Further support for the accuracy of our findings can be derived from an analogy to awakenings and movement in patients with parasomnias. Patients with parasomnias are more likely to wake up at the end of SWS, and awakening is more likely with longer durations of SWS episodes, which is more likely at the beginning of the night [52]. We did not statistically examine the proportion of SWS episodes that terminated with movement and were followed by a wake period as compared to those where just another sleep phase followed. We refrained from this analysis mainly due to the low number of awakenings following SWS movement termination. Although the movements documented in the present study did not regularly coincide with awakening, we could postulate that they reflect the lower end of the spectrum from mild arousals marked by movement to full awakening, which follows the pattern of being more likely with longer SWS duration at the beginning of the night. Furthermore, it would have been valid to extend this analysis to documenting the frequency of SWS transitions from N3 to N1/Wake and then quickly back to the deep sleep stage without movement. We did not investigate such interruptions of SWS since the focus of the present analysis was on the movement termination of SWS. However, these transitions might have contributed to those cases in our sample with highly fragmented sleep, i.e., with a high number of SWS episodes. Specifically, the prominent change from SWS to N1 and back to SWS might have led to the classification of that episode into two episodes.

### 4.2. Spectral Properties of SWS in Relation to Movement Termination

According to our data, it is unlikely that the neuronal dynamics during SWS reflect the movement propensity, since we did not find any significant relationship, only trending associations. Nevertheless, it is possible that the trends in specific frequency bands hint at underlying physiological processes that merit further exploration. According to our data, the alpha and sigma band power in the parietal region were by tendency linked to the occurrence of movement at the end of SWS. The investigated frequency ranges of alpha and sigma include the frequency range of 10 to 16 Hz, which is typical for sleep spindles. Spindles have been found to be overall slower at frontal locations as compared to posterior locations [53]. It was furthermore suggested that the exact distinction between frontal and parietal spindles is not simply explained by a difference in their frequency range, and the frequency range of these two phenomena overlaps to a great extent [53]. Although our data revealed only trends, there might be an association between movement at the end of SWS and parietal sleep spindles that warrants further investigation. Like slow waves, sleep spindles during SWS are related to sleep pressure and, therefore, reflect another correlate of sleep homeostasis [54]. Our study did not systematically investigate sleep deprivation, such that no conclusions can be drawn in this respect. Future research should investigate the occurrence of SWS termination by movement under controlled conditions of sleep deprivation.

### 4.3. Medication and SWS Terminated by Movement

Finally, the study explored the influence of medication intake on SWS termination patterns. While most medications did not show a significant effect, Sertraline emerged as a notable exception, abolishing SWS terminations with movement entirely in the assessed population. Although an additional permutation analysis comparing the number of people not showing any SWS terminated by an arousal between those taking Sertraline vs. those taking any other type of medication was significant, but this unexpected finding is limited by the small sample of patients taking Sertraline and should not be overinterpreted. However, this result underscores the need for cautious consideration of medication effects on the sleep architecture, particularly in populations with neurological disorders.

The finding is also in line with Sertraline being an effective medication to treat parasomnias that manifest as movement during NREM sleep [55]. It is also interesting to take note about a case with major depressive disorder who repeatedly experienced sleep paralysis after the onset of therapy with Sertraline [56]. While other SSRIs such as fluoxetine have been found to increase awakenings [57], Sertraline was found to have rather beneficial effects on the sleep architecture by increasing the latency to the first REM period [58]. Our data suggest that Sertraline might downregulate the system responsible for allowing movement during deep sleep, but this finding must be replicated in a larger sample taking into account the other factors such as diagnosis (epilepsy, depression).

### 4.4. Limitations

A relevant limitation of this study is that we did not examine the possibility of myoclonic phenomena, such as jerks during sleep that represent the most common type of physiological myoclonus, essential myoclonus, epileptic myoclonus, or symptomatic myoclonus [59].

Naps during the day are known to affect sleep, in the form of reducing the pressure for SWS and increasing the latency for sleep [60]. In this study, we did not control for the number of naps during the day before the experimental night.

Another limitation is that the EEG in the EMU is similar but not equivalent to standard polysomnography for sleep scoring [61]. Nevertheless, sleep studies in patients with epilepsy are usually conducted in the EMU. The EMU is a medical environment where patients are required to stay in bed all day to ensure their safety [62]. They undergo different procedures that increase their likelihood of experiencing seizures. Some EMU settings allow patients to be in a single bedroom, but other EMUs have more than one patient in a room, facilitating fewer staff to monitor more patients at a time. In Europe, 1–2 beds for monitoring are found in 37% of EMUs, 3–5 beds in 56%, and in 7% there are even 10–11 beds [62], but the number of beds per room varies. While it is plausible that SWS interruptions could arise from noise arising from sharing a bedroom with other patients, our data did not support this interpretation. However, since we did not investigate the concurrently recorded video to examine the possible disturbances from other patients that might have caused the termination of SWS resulting in movement, we cannot exclude this possible confounder. Future studies could explore this possibility.

In the analysis of the effects of individual medications, there is considerable difference in the total duration of SWS between people taking certain types of drugs (e.g., Benzodiazepines and Oxcarbazepine) as compared to controls, but these comparisons suffer from low power because of only a few people taking these drugs.

### 4.5. Future Directions

While we did not document any association between movement termination of SWS and demographic variables, epilepsy, SWS duration, SWS power spectra, and memory, several avenues for further research remain. The most interesting question for future investigations is whether there is any variable that allows to explain why some SWS episodes end with movement, while others do not. Similarly, it is of interest why a large proportion of participants did not demonstrate any SWS termination by movement. While our data suggest that certain medication might abolish the movement at the end of SWS, this finding warrants systematic replication in a larger sample.

## 5. Conclusions

In conclusion, the contributions of the present study are as follows:

- We provide valuable insights into the prevalence and distribution of the movement termination patterns of SWS.
- The overall duration of SWS was related to the number of SWS episodes that ended with movement, suggesting that these movements were related to sleep continuity.
- Furthermore, it is possible that our data provide an explanation for certain medications, such as Sertraline, being beneficial for sleep. According to our data, part of the positive action of Sertraline might be because it suppresses movements at the end of SWS.

## Figures and Tables

**Table 1:** Coefficients and significance of the logistic regression model to determine the number of SWS episodes ending with movement during the assessed night.

Factor	Estimate	Std. Error	*t*-Value	*p*-Value
age	−0.002	0.018	−0.100	0.921
sex	0.085	0.305	0.279	0.781
BDI depression score	0.017	0.020	0.837	0.406
DMEQ chronotype score	0.017	0.016	1.063	0.292
Total duration of SWS	0.022	0.004	5.638	0.001 *
Age of seizure onset	0.017	0.015	1.111	0.271
DDD ASM	0.211	0.161	1.308	0.195
DDD Psychoactive medication	−0.357	0.283	−1.261	0.212
Focal epilepsy vs. …				
No epilepsy	0.801	0.617	1.298	0.199
Undetermined seizure type	−0.047	0.463	−0.101	0.920
Generalized seizures	0.197	0.423	0.466	0.643

BDI: Beck Depression Inventory; DMEQ: German version of the Morningness–Eveningness Questionnaire; SWS: slow-wave sleep; DDD: defined daily dose; ASM: Antiseizure medication; Std. Error: standard error; * significant result; note that the analysis excluded the 18 patients where BDI and/or DMEQ information was missing.

**Table 2:** Results of the logistic regression model to determine the number of movement terminations of SWS that occurred during the assessed night. Only results with *p* < 0.100 are shown.

Factor	Estimate	Std. Error	*t*-Value	*p*-Value
Frontal right delta	1.182	0.707	1.672	0.098
Occipital left delta	0.722	0.407	1.777	0.079
Temporal right theta	−7.496	4.252	−1.763	0.082
Parietal left alpha	16.408	7.827	2.096	0.039
Frontal right sigma	19.973	11.734	1.702	0.093
Parietal left sigma	24.188	9.655	2.505	0.014
Parietal right sigma	−20.8514	8.884	−2.347	0.021

**Table 3:** Effect of individual medications and groups of medications on the percentage of SWS that ended with movement.

		Medication	All Others	vs. All Others		vs. No Medication	
Medication	*n*	Mean (SD)	Mean (SD)	*t*-Value	*p*-Value	*t*-Value	*p*-Value
Valproate	7	0.27 (0.39)	0.23 (0.28)	−0.206	0.843	−0.406	0.697
Levetiracetam	32	0.24 (0.31)	0.24 (0.29)	−0.033	0.974	−0.494	0.623
Lacosamide	7	0.39 (0.42)	0.22 (0.28)	−1.037	0.337	−1.142	0.290
Carbamazepine	7	0.1 (0.19)	0.25 (0.30)	1.868	0.097	1.179	0.261
Oxcarbazepine	9	0.3 (0.36)	0.23 (0.28)	−0.567	0.585	−0.756	0.466
Any ASM	51	0.24 (0.30)	0.24 (0.29)	0.054	0.957	−0.517	0.607
Any APM	4	0.25 (0.29)	0.24 (0.29)	−0.095	0.930	−0.314	0.771
Any z-drug	4	0.10 (0.21)	0.24 (0.29)	1.273	0.280	0.856	0.436
Sertraline	4	0 (0)	0.25 (0.29)	8.003	<0.001 *	4.224	<0.001 *
Any SSRI	9	0.22 (0.27)	0.24 (0.29)	0.224	0.827	−0.142	0.890
Any ADM	14	0.26 (0.27)	0.23 (0.30)	−0.365	0.720	−0.684	0.500

n = number of patients taking that drug/drug within that group of drugs; ASM: antiseizure medication; APD: antipsychotic medication; z-drug: Benzodiazepines; SSRI: selective serotonin reuptake inhibitors; ADM: antidepressive medication; * significant result.

**Table 4:** Effect of individual medications and groups of medications on the total duration of SWS in minutes.

		Medication	All Others	vs. All Others		vs. No Medication	
Medication	*n*	Mean (SD)	Mean (SD)	*t*-Value	*p*-Value	*t*-Value	*p*-Value
Valproate	7	78.40 (19.90)	71.44 (40.34)	−0.802	0.440	−1.007	0.318
Levetiracetam	32	71.09 (30.62)	72.41 (43.14)	0.171	0.865	−0.494	0.623
Lacosamide	7	83.54 (33.35)	71.03 (39.62)	−0.941	0.376	−1.461	0.184
Carbamazepine	7	53.37 (23.47)	73.46 (39.87)	2.040	0.071	1.059	0.316
Oxcarbazepine	9	104.01 (80.13)	68.57 (31.06)	−1.317	0.223	−1.478	0.176
Any ASM	51	77.53 (44.41)	65.36 (31.09)	−1.556	0.123	−1.757	0.083
Any APM	4	90.70 (48.03)	71.13 (38.84)	−0.804	0.477	−1.094	0.349
Any z-drug	4	95.27 (33.06)	70.92 (39.25)	−1.429	0.239	−1.826	0.153
Sertraline	4	69.43 (30.33)	72.07 (39.64)	0.168	0.876	−0.346	0.749
Any SSRI	9	66.90 (28.43)	72.50 (40.23)	0.536	0.602	−0.,281	0.784
Any ADM	14	68.84 (36.42)	72.51 (39.82)	0.343	0.736	−0.455	0.655

n = number of patients taking that drug/drug within that group of drugs; ASM: antiseizure medication; APD: antipsychotic medication; z-drug: Benzodiazepines; SSRI: selective serotonin reuptake inhibitors; ADM: antidepressive medication.

## Data Availability

The data generated for this study are available in the Supplementary Data Section S1 (S1-data.xls). Due to the sensitive nature of the data, the raw EEG datasets generated for this study can be made available upon reasonable request and after application and approval by the relevant ethical authority.

## Supplementary Materials

The following supporting information can be downloaded at https://www.mdpi.com/article/10.3390/brainsci14050493/s1. The file, S1-data.xls, contains the data generated and analyzed in the present study.
